# Enhancer network revealed by correlated DNAse HS states of enhancers

**DOI:** 10.1186/1753-6561-6-S6-P28

**Published:** 2012-10-01

**Authors:** Justin Malin, Radhouane Aniba, Sridhar Hannenhalli

**Affiliations:** 1 Center for Bioinformatics and Computational Biology, University of Maryland, College Park, MD, USA

## Background

Mammalian gene regulation is often mediated by distal enhancer elements. Despite this, mechanistic investigations of correlated expression have thus far been focused on proximal promoter regions, mainly because distal enhancers are not known for a vast majority of genes. However, distal enhancers of co-regulated genes are likely to have correlated activity.

## Materials and methods

Using P300-bound regions as putative enhancers [1] and using cell-type-specific DNAse hypersensitivity (HS) at these enhancers as an operational definition of enhancer activity, here we perform a detailed investigation of enhancer activity correlation across 15 cell types, followed by analysis of mechanistic underpinnings and functional consequences of correlated enhancer activity. We initially identify pairs of highly correlated enhancers from the same (*cis*) and from different chromosomes, after accounting for HS autocorrelation affecting *cis*-pairs. Using non-parametric tests and controlling for dependencies, highly correlated pairs are compared with background pairs for: enrichment of co-occurring binding motifs; for correlated gene expression across the 15 cell samples sourced for HS data; for shared gene function; for evidence of interactions between shared enhancer-binding transcription factors (TFs) and chromatin-modifying enzymes; and for Hi-C evidence of pair co-localization. The relationship between correlated enhancers now established, we conclude by scaling this pairs perspective to the building and validating of an enhancer network.

## Results

We find that correlated enhancers tend to share common TF-binding motifs, and that several chromatin modification enzymes preferentially interact with these TFs. We show that we can predict correlated enhancers with 73% accuracy based only on the presence of shared motifs for specific TFs. Also, genes near correlated enhancers have correlated expression and share common function. Correlated enhancers coincide with spatially proximal genomic regions assayed by Hi-C in two different cell types. Finally we construct an enhancer network based on shared motifs and correlated activity, and show its high overlap with biological processes and pathways.

## Conclusions

Overall, our analysis suggests that functionally linked genes may be co-regulated by distal enhancers whose activities are regulated by common sets of TFs and mediated by both 3D chromatin structure as well as chromatin modification enzymes. Our work represents the first investigation of enhancer networks based on correlated activity across multiple cell types.

## References

[B1] Visel A Blow MJ Li Z Zhang T Akiyama JA Holt A Plajzer-Frick I Shoukry M Wright C Chen F Afzal V Ren B Rubin EM Pennacchio LA ChlP-seq accurately predicts tissue-specific activity of enhancers Nature 2009 457 854 858 10.1038/nature07730 19212405 PMC2745234

